# Automated fiducial marker detection and localization in volumetric computed tomography images: a three-step hybrid approach with deep learning

**DOI:** 10.1117/1.JMI.8.2.025002

**Published:** 2021-04-28

**Authors:** Milovan Regodić, Zoltan Bardosi, Wolfgang Freysinger

**Affiliations:** a Medical University of Innsbruck, Department of Otorhinolaryngology, Innsbruck, Austria; b Medical University of Vienna, Department of Radiation Oncology, Vienna, Austria

**Keywords:** image-guided surgery, fiducial markers, convolutional neural network, open-set recognition, phantom models, virtual computed tomography

## Abstract

**Purpose:** Automating fiducial detection and localization in the patient’s pre-operative images can lead to better registration accuracy, reduced human errors, and shorter intervention time. Most current approaches are optimized for a single marker type, mainly spherical adhesive markers. A fully automated algorithm is proposed and evaluated for screw and spherical titanium fiducials, typically used in high-accurate frameless surgical navigation.

**Approach:** The algorithm builds on previous approaches with morphological functions and pose estimation algorithms. A 3D convolutional neural network (CNN) is proposed for the fiducial classification task and evaluated for both traditional closed-set and emerging open-set classifiers. A proposed digital ground-truth experiment, with cone-beam computed tomography (CBCT) imaging software, is performed to determine the localization accuracy of the algorithm. The localized fiducial positions in the CBCT images by the presented algorithm were compared to the actual known positions in the virtual phantom models. The difference represents the fiducial localization error (FLE).

**Results:** A total of 241 screws, 151 spherical fiducials, and 1550 other structures are identified with the best true positive rate 95.9% for screw and 99.3% for spherical fiducials at 8.7% and 3.4% false positive rate, respectively. The best achieved FLE mean and its standard deviation for a screw and spherical marker are 58 (14) and 14 (6)  μm, respectively.

**Conclusions:** Accurate marker detection and localization were achieved, with spherical fiducials being superior to screws. Large marker volume and smaller voxel size yield significantly smaller FLEs. Attenuating noise by mesh smoothing has a minor effect on FLE. Future work will focus on expanding the CNN for image segmentation.

## 1 Introduction

Fiducial markers are used for reliable and accurate patient registration in image-guided interventions. Such surgical interventions are performed during the placement of both a cochlear implant into the inner ear^1^ and electrodes for deep brain stimulation to treat patients with Parkinson’s disease and essential tremor.^2^ Markers are usually attached to the skin or screwed into the bone, with the latter providing greater accuracy at the cost of invasiveness.^3^ A recent method exploits spherical markers placed inside the nasal cavity (nasopharynx) and could be automatically localized by their internal magnetic sensors.^4^ Experiments with phantoms show that the advantageous marker positioning in the head provides a feasibility of submillimetric accuracy for the surgeries of the lateral skull base.^4^ Following the placement of fiducial markers and recording the patient’s pre-operative images, rigid registration is used to establish medical navigation by computing the optimal transformation between the fiducial points in the image and in the physical location of that patient during surgery. The optimal transformation can be found by minimizing the sum of the squared distances between each corresponding pair as shown by Horn.^5^ The square root of the mean of this squared distance is often referred to as fiducial registration error (FRE). The FRE, as pointed by Fitzpatrick,^6^ could indicate whether or not the registration process is functioning correctly, but, if the process is functioning correctly, then this quantity should not be used to determine the accuracy of patient registration. A more reasonable measure of the registration error is at the (surgical) point of interest referred to as target registration error (TRE).^6^ It has been shown that both the FRE and the TRE are dependent on the error in identifying the correct location of the fiducials, called fiducial localization error (FLE).^7^^,^^8^ The FLE is defined as the distance between the real and measured fiducial point. Thus, reducing FLEs both in the image and physical space can contribute to improving the registration accuracy.

Attempts with automated detection algorithms to localize fiducial markers in image space are known.^9^^–^^21^ As demonstrated in clinical interventions,^1^^,^^19^ automating this task can not only provide reproducible results, reduce human errors, and shorten intervention time but also lead to lower FLEs. Wang et al.^9^ proposed to automatically detect and localize cylindrical-shaped markers using two-dimensional (2D) morphological operations and the centroid calculation. Gu and Peters^11^ improved on that idea using the set of 3D morphological operations for detection and the intensity weighted centroid for localization of cylindrical markers. Chen et al.^12^ proposed to detect and localize the centroid point of cylindrically shaped markers that are rigidly attached to the patient’s skull using edge detection and curve fitting among other steps, whereas Tan et al.^13^ using a 2D template modeling but without exercising any localization approach. The authors^12^^,^^13^ reported a high detection rate without disclosing the exact marker type and dimensions. Fattori et al.^17^ utilized a set of surfaces extracted using Marching Cubes^22^ for the fiducial intensity levels in the CT image to detect aluminium spheres (1-cm diameter) used in optical tracking. The fiducial point is estimated as the centroid of surface vertices. Wang and Song^15^ and Bao et al. (2019)^20^ achieved high detection and localization accuracies with their methods for localizing the centers of adhesive markers attached to the patient’s skin. The used IZI multi modality markers (IZI Medical Products, MD) have a thickness of 3.5 mm, outer diameter 15 mm, and inner diameter 5 mm. The same marker, among others with cylindrical and spherical shapes, were employed in a universal semi-automatic solution by Nagy et al.^18^ The localization method utilizes an a priori fiducial model (with known actual point) that is co-registered to a local region of interest (manually marked) containing the fiducial marker in the image.

This prior work with automated detection is based on categorizing markers from other structures in the image using either hand-crafted features (e.g., intensity thresholds, the distance between markers or marker volume) ^9^^,^^11^^,^^17^ or low-level features such as a spherical shape.^12^^,^^13^^,^^15^^,^^20^ The hand-crafted features are subject to failure due to the fact that medical images are of discrete nature and image segmentation is often not completely accurate. The low-level features were shown to be optimal for detecting adhesive markers, which can be used with larger volumes due to their non-invasiveness and thus making them more distinguishable and easier to detect compared to other structures in images. Nonetheless, as mentioned, the screws implanted into bone are more precise and used for frameless interventions requiring high accuracy, for instance, in robotic cochlear implantation.^1^ Smaller screw dimensions are preferable to reduce surgical invasiveness. The centroid point, however, is suboptimal for surgical screws as it is biased away from the screw head. Moreover, the centroid calculation might impair precision resulting from deformed segmented objects. Zheng et al.^16^ resolved localization for the screw fiducials by using a pose estimation technique. However, the detection of fiducial markers in the image is done semi-automatically.

In this work, we realize a fully automated algorithm by combining and improving Gu and Peters^11^ and Zheng et al.^16^ works so that automated detection—*segment and classify fiducial markers*—and automated localization—*estimate fiducial position*—are unified and work for both tiny surgical screws and spherical fiducials. In contrast to prior work, marker classification is investigated with deep convolutional neural networks (CNNs), which are able to learn various aspects of images at different feature levels.^23^ Identification of markers versus other structures is evaluated with two traditional closed-set and two emerging open-set classifiers employed during CNN training. To assess the detection rate of those approaches, the independent CT images unseen during training are tested.

In this paper, several approaches for image FLE estimation are reported. Some authors^12^^,^^15^^,^^16^^,^^18^^,^^20^ assess localization accuracy by verifying the measured position with the position detected by an individual. It was experimentally shown that the individuals are conditioned to deviate from the real ground-truth positions.^24^ A more reliable ground-truth measure to establish a controlled environment is reported with phantoms using a coordinate measuring machine,^25^^,^^26^ accurate laser tracking measurement,^17^ or intra-modal registrations of two different CT datasets with the same fiducial configuration.^26^ However, as correctly noticed,^26^ these methods may inflate FLEs, resulting in higher values due to uncertainties in image registration and geometrical distortions, which change fiducial configuration. Unlike those approaches, we opted for a fully virtual digital experiment to establish a ground-truth measure to get the best estimates for FLE in the image.^24^ This approach utilizes CONRAD (v. 1.1.0),^27^ an open-source software framework for cone-beam CT (CBCT) imaging, which provides full control of projection and reconstruction parameters.

This paper is an improved (mostly in the marker classification part) and extended version of the contribution presented at the SPIE Medical Imaging 2020 conference.^21^ For readers interested in reproducing our results, parts of our code and other materials used in this paper can be obtained in a Github repository (https://github.com/mregodic/FiducialMarkers).

## 2 Materials and Methods

**Fig. 1:**
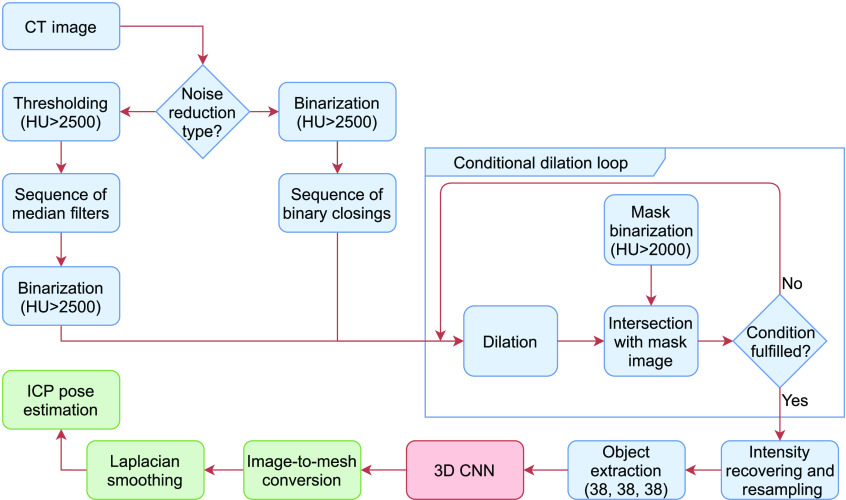
The algorithm workflow with thresholding and morphological operations for image segmentation (blue) followed by a 3D convolutional neural network (CNN) for classification (purple) and finally fiducial localization (red).

Figure 1 shows the workflow of the algorithm, which is described in detail in Secs. 2.1, 2.2 and 2.3, and 2.4 describes the virtual phantom.

### 2.1 Marker Segmentation

Compared to Gu and Peters,^11^ our approach with segmentation does not include the top-hat (TT) grayscale morphological operation to determine the histogram values of the markers. The TT operation can be avoided if the Hounsfield units (HU) are already known for the marker material in the CT image (e.g., titanium is ∼3000  HU). Also, the TT is very computationally expensive in grayscale compared to binary morphology as the pixel values (e.g., finding the minimum and maximum) are compared for integers, one pixel at a time. The binary opening operation for noise reduction is optimized with a sequence of grayscale median filters or better computational performance^28^—a binary dilation followed by a sequence of binary erosions (binary closing). This optimization will better preserve the original image data and is much less sensitive to discretization effects occurring in large voxel sizes (e.g., $0.5\times 0.5\times 0.8\;{\mathrm{mm}}^{3}$) or in smaller markers (e.g., tiny surgical screws).

Following image thresholding and noise reduction, conditional dilation can recover deformed markers with a series of dilations intersected with a mask image to limit dilation results to the inside of the region of interest.^11^ The stop conditions are (1) no change in the number of different voxels between iterations; (2) a maximum number of iterations reached; and we added one more criterion (3) if the number of different voxels between iterations increases (or, if the number of different voxels does not monotonically decrease until the difference is zero). Although not a perfect condition, (3) can help stop unnecessary dilations of noise or other structures.

The resulting binary image is intersected with the original image to recover intensity values. The segmented objects are extracted on the criterion that their pixels are fully connected (26-connectivity for a three-dimensional image) with non-zero values.

**Fig. 2:**
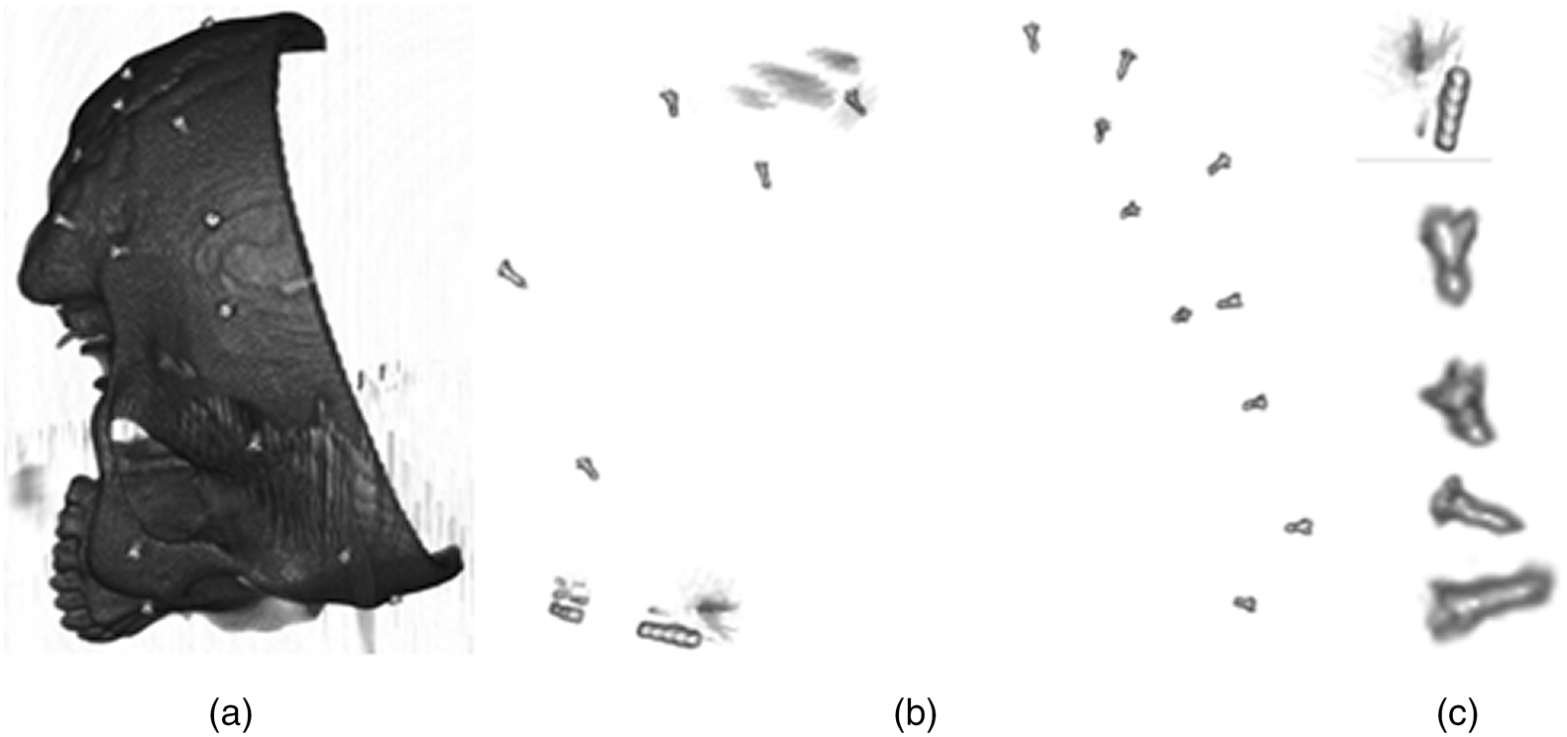
(a) Visualization of the right side of a skull phantom in a virtual CBCT image generated in CONRAD. The bright spots are screw fiducials ($2\times 3\;{\mathrm{mm}}^{2}$) in the image with the voxel size 0.5×0.5×0.5  mm. (b) The segmented scene from (a) using the presented approach. The scene is magnified and rotated differently compared to (a). (c) The magnified samples from (b) with the first from top being a non-marker structure and the rest screws.

**Fig. 3:**
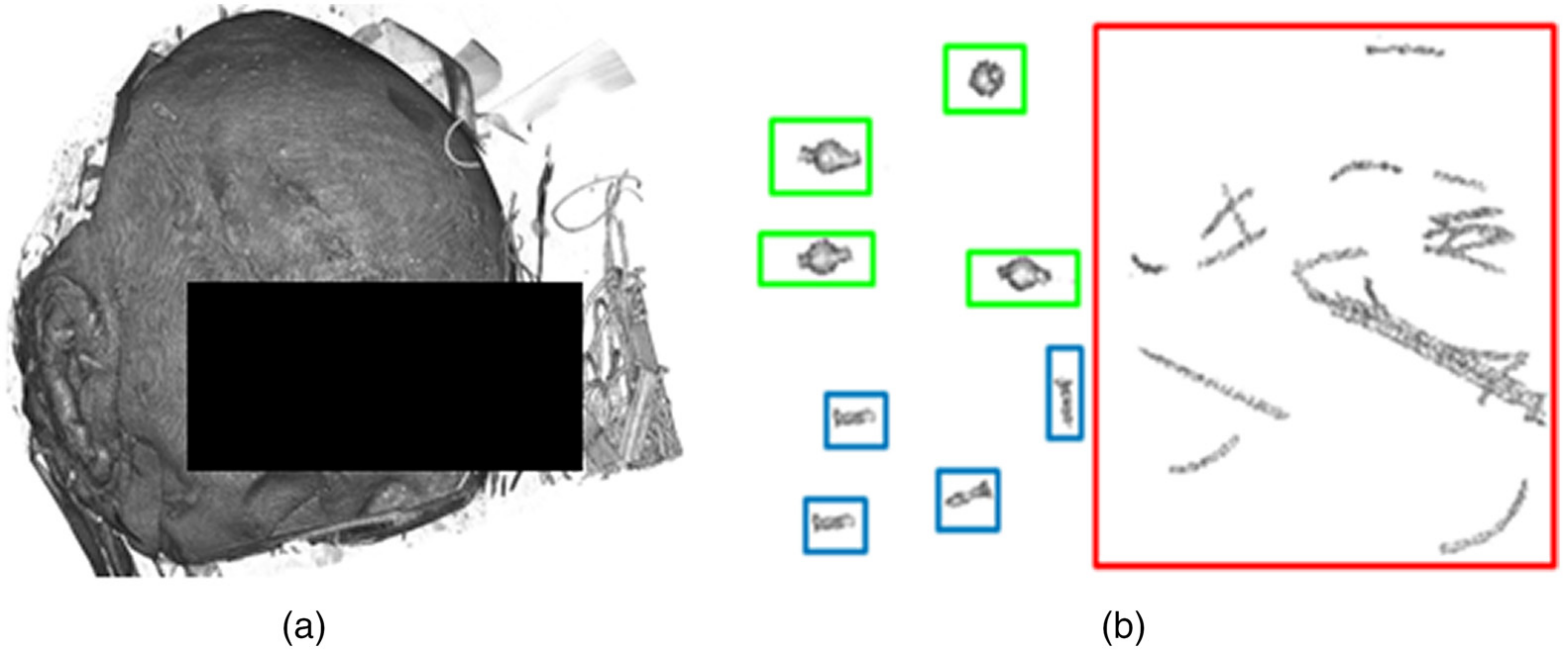
(a) Visualization of a head (anatomical specimen) with implanted titanium screw and spherical markers. (b) Segmented head (a) using the presented approach, and inside added colored boxes: screws in blue, spherical fiducials in green, and other structures in red.

Examples of segmented images using this method are shown for a simulated CBCT in Fig. 2 and for a human anatomical specimen in Fig. 3.

#### 2.1.1 Segmentation implementation details

The segmentation model is implemented in the C++ programming language using ITK^29^ library. The algorithm was run on CPU Intel Core i7-7700K 4.2 GHz, RAM 16 GB and GPU NVIDIA GeForce GTX 1050 (8 GB GPU RAM).

### 2.2 Marker Classification

#### 2.2.1 Convolutional neural network

As shown in Figs. 2 and 3, the segmentation is imperfect, and in addition to noise not being completely removed, some non-marker structures may appear. To automatically select markers, a 3D CNN is trained that accepts an image of the segmented object as input, pulls through the series of convolutional layers to learn a sense of three-dimensional features and outputs scores for each class (e.g., screw, spherical fiducial, or background). In general, CNNs are deep learning algorithms that are able to capture and relate features in images by nonlinear transformations in a multi-layer structure.^23^ These transformations extract both low-level features (e.g., edges, curves, and lines) and semantic features related to visual representation and object recognition.

#### 2.2.2 CNN architecture

**Fig. 4:**
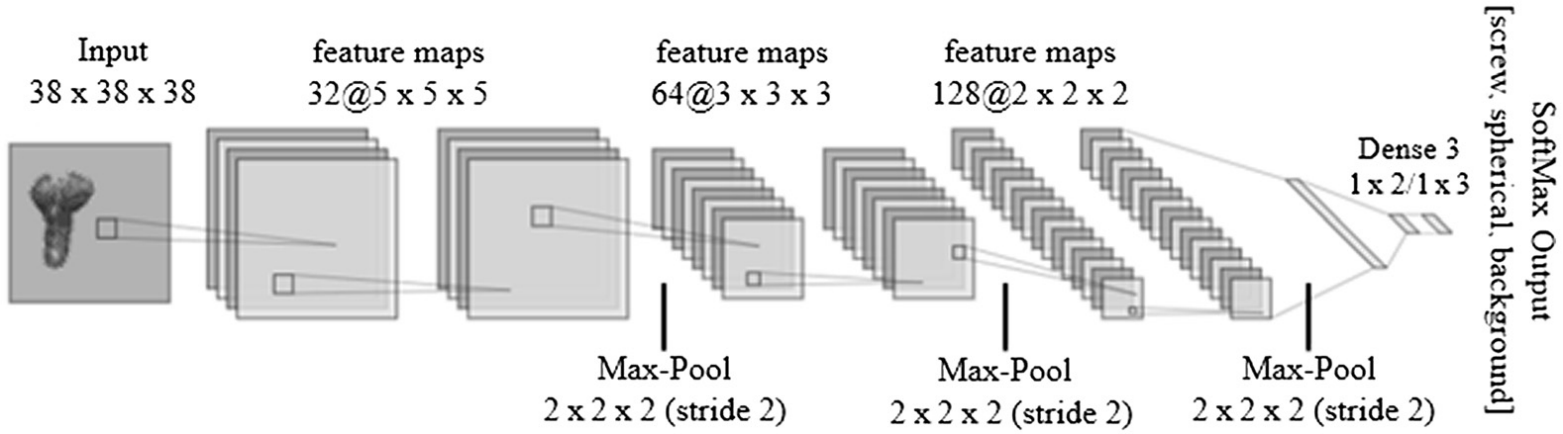
A 3D CNN for classification of fiducial markers. The class (e.g., screw, spherical fiducial, or background) to which the input image belongs is determined at the output of a fully connected neural network at the neuron with the highest value.

CNNs were first introduced by LeCun et al.^30^ in the late 90’s. In this groundbreaking paper, among other valuable things, a LeNet deep network architecture is suggested for the classification of handwritten digits in the MNIST dataset. We found that an extended version of this architecture works sufficiently well (Fig. 4). The extended model consists of six layers formed in three blocks with two layers stacked before batch normalization and subsampling (Max-Pool). Feature maps and their kernel size in a convolutional layer are progressively adapted with the first block 32 and 5×5×5, the second block 64 and 3×3×3, and the third block 128 and 2×2×2. The convoluted features are inputs to a fully connected network followed by a softmax output function at the end (or sigmoid in the case of binary classification) to produce a probability distribution over a set of known classes.

#### 2.2.3 CNN classifiers

A CNN model learns and optimizes samples from known classes. However, in addition to screw and spherical fiducials, our network can be fed with images of the segmented structures that do not belong to any fiducial class and should be recognized as outliers. In the deep learning field, the former samples can be categorized as knowns and the latter as unknowns. Traditionally, the unknown samples are treated with rejections under a certain threshold of the activation function or training the network with an additional background class containing a diverse set of unknown samples. The former approach assumes that unknown samples will have small probabilities compared to knowns. However, it is reported that uncertainty of unknowns is insufficient as the networks can be biased toward a particular class^31^ and fooled with unknown samples achieving high probabilities.^32^ On the other hand, although more effective, training the network with known unknown samples belonging to a background class can only represent a closed set of the unknowns. Attempts with emerging open-set classifiers incline to address this gap in the field.^31^ Among these methods, we highlight recent entropic openset and objectosphere approaches,^33^ which tend to have sufficiently good results and outperform others empirically. These methods modify the loss function to produce the network of a smaller feature magnitude (Euclidean norm), ‖F(x)‖, for background samples. For network input x, F(x) represents an activation value at the output of the neurons in the penultimate layer that feeds into the final softmax layer. In particular, entropic openset loss $J_{E}$ indirectly affects the magnitude of unknowns by modifying the softmax scores for known class c∈C:

$$J_{E}(x)=\{\begin{array}{ll} -\log S_{c}(x) & \text{if}\;x\in \text{known samples}\\ -\frac{1}{C}\overset{C}{\underset{c=1}{\sum }}\log S_{c}(x) & \text{if}\;x\in \text{unknown samples} \end{array},$$ (1)

where $S_{c}$ is the standard softmax function and known/unknown samples are in our case fiducials/not-fiducials. Objectosphere loss $J_{R}$ increases even further this margin by maximizing the magnitude of knowns and minimizing the magnitude of unknowns at the same time:

$$J_{R}(x)=J_{E}+\lambda \{\begin{array}{ll} \max{\left(\xi -\|F(x)\|,0\right)}^{2} & \text{if}\;x\in \text{known samples}\\ {\left\|F(x)\right\|}^{2} & \text{if}\;x\in \text{unknown samples} \end{array},$$ (2)

where ‖F(x)‖ is feature magnitude of activation values in the penultimate layer of the network, ξ is a predefined margin constraint for the minimum magnitude of known samples, and λ balances two elements of the error.^33^ Furthermore, the general idea of the objectosphere loss is to threshold the feature magnitudes multiplied with softmax probabilities $\left\|F(x)\|\cdot S_{c}(x\right)$ instead of just the softmax probabilities $S_{c}(x)$.^34^ To minimize incorrect detection of adversarial structures as fiducial markers, we evaluate which of the mentioned traditional and open-set classifiers performs best.

#### 2.2.4 Training/validation dataset

The training dataset was constructed from segmented CT images using the described method, with one of the authors verifying the correctness of the automatic algorithm. A total of 210 screw and 22 spherical fiducials were segmented from 15 CT images of three human anatomical specimen heads (13 screws, four spherical fiducials), one porcine head (four spherical fiducials), and 11 phantoms (197 screws, 14 spherical fiducials). The slice thickness in images varied from 0.4 mm up to 1 mm. The adversarial non-marker structures resulting during the process mentioned above are considered segmentation errors and added to the unknown sample dataset. Additional unknown samples were introduced by thresholding images (HU>1500), performing one morphological binary opening, and extracting non-marker objects. Fiducials generated in CONRAD were also added to improve the detection of datasets used for the localization assessment (see Sec. 3.2). As pointed in the literature,^35^ we augment the available data by multiple random rotations and translations to increase network performance. This resulted in a total dataset containing 4000 images of fiducial markers, in equal proportions for screws and spherical fiducials, and 3462 images of various unknown structures. For class balance, 3000 and 1500 images for multi-class and binary classifiers were randomly selected from the background population. The images in the dataset were resampled to 0.33 mm isotropic resolution, scaled to 0-255 (float) range using min-max linear intensity transformation and randomly divided into 75% training and 25% validation datasets. The scaling was used to reduce the effect of intensity variations in CT images and was done per segmented object using the whole range inside the region of interest. Further, as a consensus to improve training speed and classification accuracy,^32^^,^^35^ the network input was standardized with a mean of 0 and a standard deviation of 1 based on the training dataset values.

#### 2.2.5 CNN implementation details

**Fig. 5:**
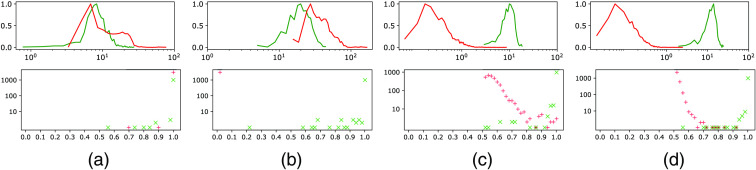
The figures in upper are normalized histograms of feature activation magnitudes from a penultimate layer of multi-class classifiers with a logarithmic horizontal axis and normalized frequency (divided by the max value) in a vertical axis. And in bottom histograms of final softmax probabilities with a logarithmic vertical axis. In (a) softmax thresholding; (b) background class; (c) entropic openset; and (d) objectosphere. In general, it can be observed that the magnitudes and probabilities of the unknown samples (red line/+) tend to have lower values than the magnitudes and probabilities of the known samples (green line/x). Looking at the histogram of probabilities (in the bottom figures): (a) softmax thresholding will lead to incorrectly categorize almost all unknown samples as knows even with a very high threshold; (b) introducing the background class shows an excellent separation between unknown and known samples; (c) entropic openset; and (d) objectosphere show that a trade-off with a high threshold is needed to discriminate between unknown and known samples. However, as pointed out,^33^ when in addition looking at the histograms of magnitudes (in the upper figures): (c) entropic openset shows a much better separation between unknown and known samples compared to (a) and (b), and by using (d) objectosphere it was possible even further to increase this margin.

**Fig. 6:**
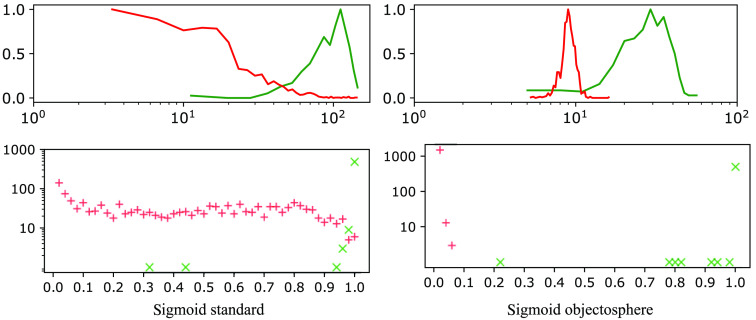
Response of dedicated screw-binary classifiers with bottom histograms representing sigmoid probabilities.

**Fig. 7:**
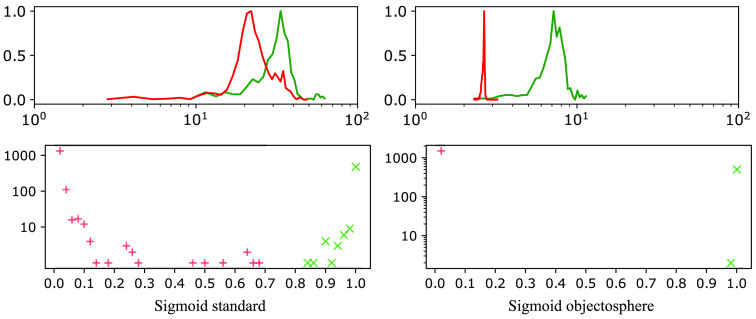
Response of dedicated spherical fiducial-binary classifiers with bottom histograms representing sigmoid probabilities.

Our network was implemented using Keras (v 2.3.1) and TensorFlow (v. 2.1.0) deep learning libraries developed in Python. Binary and categorical cross-entropy losses were used. In the case of binary classification, the final scores were calculated using the standard sigmoid activation. To minimize loss function, Adamax—the modified version of adaptive momentum estimation (Adam)^36^ optimizer was used with initial learning rate of 0.001. Four binary and four multi-class models were trained with 95 mini-batches combined with dataset shuffling over 1000 epochs. After each epoch, the model was run on the validation dataset and validation accuracy and loss were calculated. To avoid overfitting, early stopping occurs if there is no improvement in validation loss after 35/70 epochs. The minimum validation loss was achieved with validation accuracy during training larger than 99%, which occurred for multi-class models softmax thresholding, background class, entropic openset, and objectosphere after epochs 278, 165, 210, and 393, respectively. And for binary models sigmoid standard/objectosphere after epochs 93/108 in screw and 131/276 in spherical fiducial. The objectosphere’s hyperparameter ξ was set to 15. The models were trained and run on a Windows 10 machine utilizing one NVIDIA GeForce GTX 1050 (8 GB GPU RAM). The histogram of probabilities and magnitudes of the trained classifiers for fiducials in the validation dataset and other structures in the training dataset is shown in Figs. 5, 6, and 7 using a similar representation as in Dhamija et al.^33^

### 2.3 Marker Localization

**Fig. 8:**
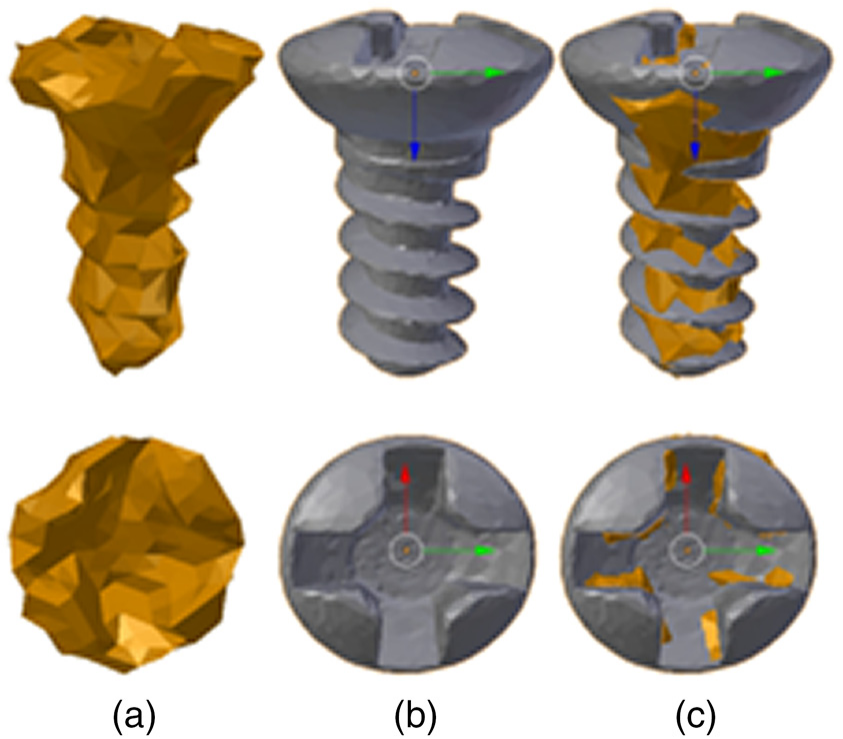
Samples of screw models with screw body and cross-head in upper and bottom figures. (a) A mesh model of the segmented screw in a virtual CBCT image. (b) A mesh model of the reference screw used for the alignment. The bottom of the screw at the cross-head section is defined as a point of reference (marked in x, y, and z axes). (c) Coregistered segmented screw model (a) to the reference model (b).

To localize positions of detected markers, we used the approach from Zheng et al.^16^ that is based on estimating a 3D relative pose between detected markers and reference marker mesh models. The reference model has a fiducial point of interest marked in the center of the spherical fiducial or on the cross-section of the screw head [Fig. 8(b)]. As proposed, the iterative closest point (ICP) algorithm^37^ was used to align the two mesh models. Once the models are aligned [Fig. 8(c)], a rigid transformation applied on the reference point of interest calculates the fiducial point in the image space.

It should be noted the downside of this approach that the ICP algorithm needs a good initial transformation estimate to find the best alignment. In our case, we do not use a pure spherical fiducial—where an identity rotation would be enough for ICP initialization, but rather the union of a sphere and cylinder. We workaround by running the algorithm multiple times for different orientations of the reference model and considering the alignment with the closest distances between the two point sets. The applied rotations were around the y-axis in Euler’s angles from 0 deg to 180 deg in steps of 30 deg.

The 3D surfaces or mesh models of the segmented markers were constructed using the Flying Edge algorithm.^38^ For our data, we experienced that this algorithm is significantly faster and provides more smooth surfaces compared to the Marching Cubes.^22^ In addition, Laplacian smoothing^39^ was applied to the mesh of detected markers prior to running the ICP to attenuate imaging noise and distribute vertices more evenly with limited shrinkage and was studied with respect to FLE localization accuracy (see Sec. 3.2). Examples of the used mesh models are shown in Fig. 8.

### 2.4 Virtual Phantom

**Fig. 9:**
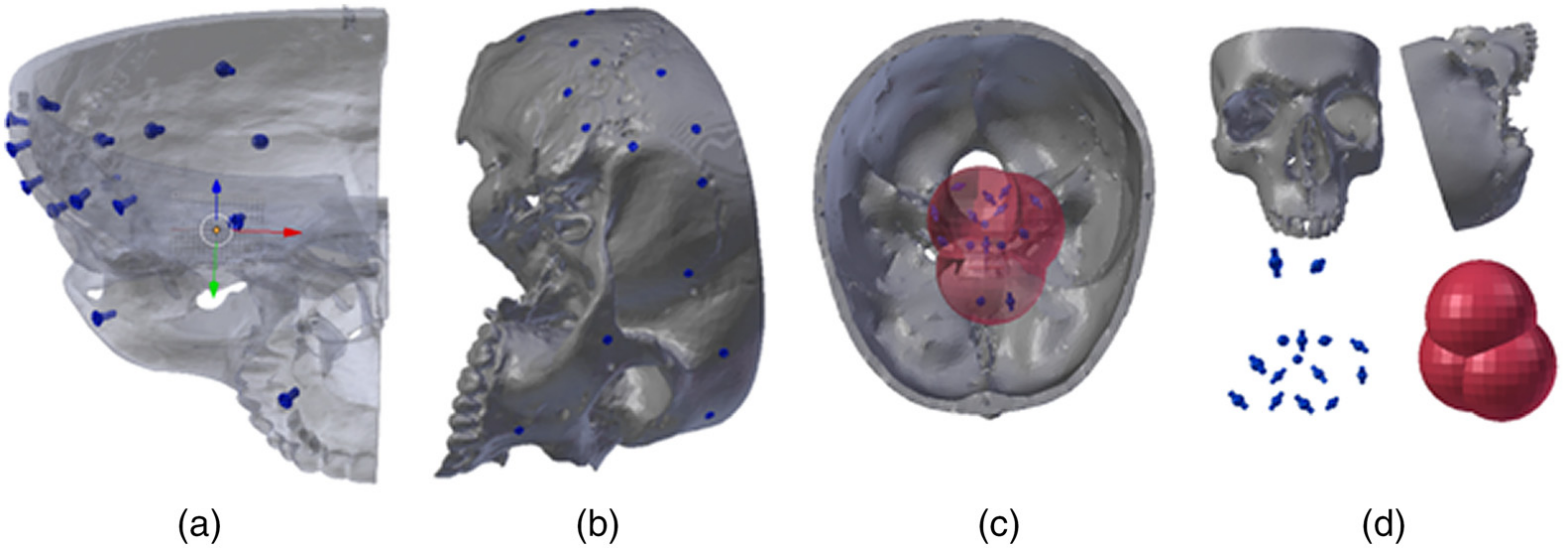
Example of scene models used to generate CBCT images in CONRAD. (a) and (b) Frontal (transparent) and lateral view of the modelled skull phantom. The model contains screws inserted till the head into the body of the skull mesh. (c) A view from the top on the skull phantom with spherical markers (blue) embedded into a soft tissue (transparent red object) in the vicinity of the nasal cavity. (d) Parts of scene in (c): frontal and lateral view of the skull phantom, spherical fiducials and red structure representing soft tissue.

**Fig. 10:**
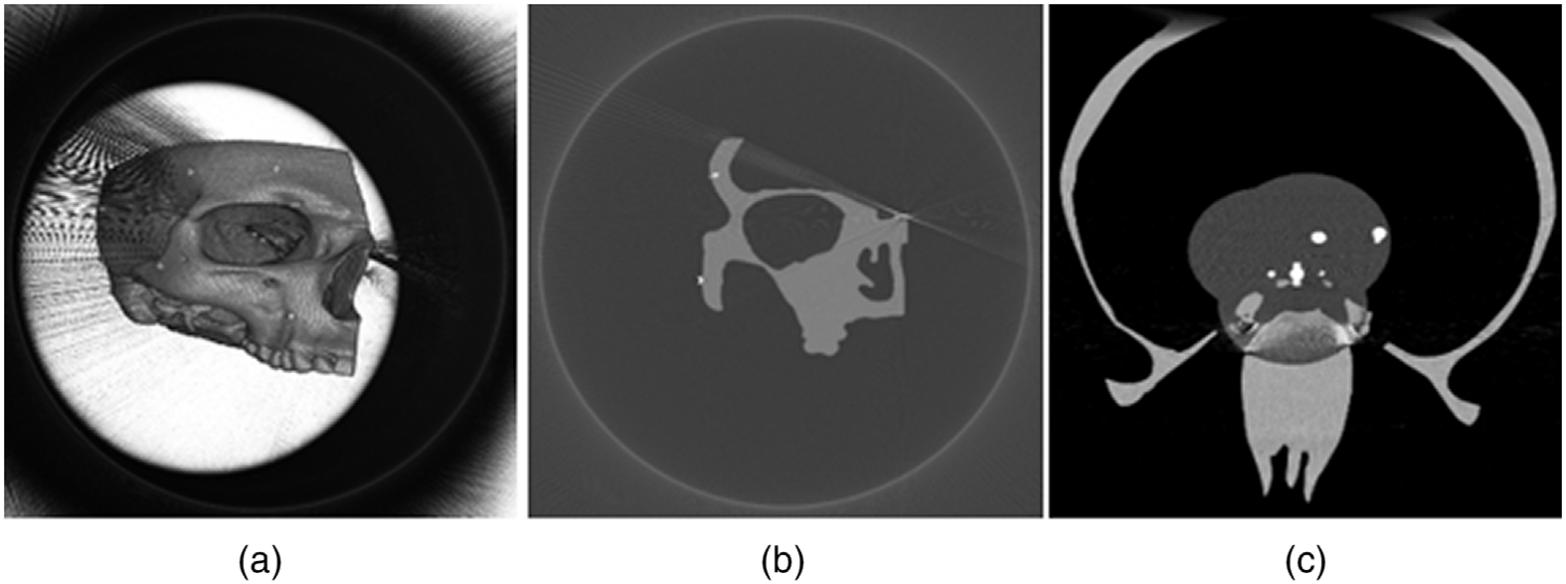
CBCT images of a virtual skull phantom generated with CONRAD (note the different windowing in viewers). (a) A 3D reconstructed image with 15 implanted screws into the skull (bright spots on the skull surface). (b) A slice in the coronal direction containing two implanted screws into the skull. (c) A slice in the axial direction with spherical fiducials placed in the soft tissue in the vicinity of the nasal cavity.

Images from CONRAD were generated from multiple 3D mesh scenes (e.g., a skull phantom scene and screw scene) created in Blender (v. 2.79, https://www.blender.org/). The mesh models of markers with different sizes and shapes were combined with the phantom mesh. The origin of the mesh is placed at the center of the scene [see Fig. 9(a)]. The original screw marker mesh was generated from a real screw ($1.8\times 3\;{\mathrm{mm}}^{2}$) imaged with a Scanco vivaCT 40  μCT (Scanco Medical AG, Switzerland) device at 70 kV.^24^ The spherical marker mesh was designed in-house. A CT image of a plastic skull phantom (scanned with Siemens CT at 120 keV with resolution of $0.33\times 0.33\times 0.40\;{\mathrm{mm}}^{3}$) was used to generate the skull mesh with 3D Slicer (v. 4.10.2, https://www.slicer.org/). Figure 9 shows examples of the phantom scenes and Fig. 10 of generated CBCT images using those scenes.

## 3 Results

### 3.1 Evaluation of the detection method

#### 3.1.1 Testing dataset

**Fig. 11:**
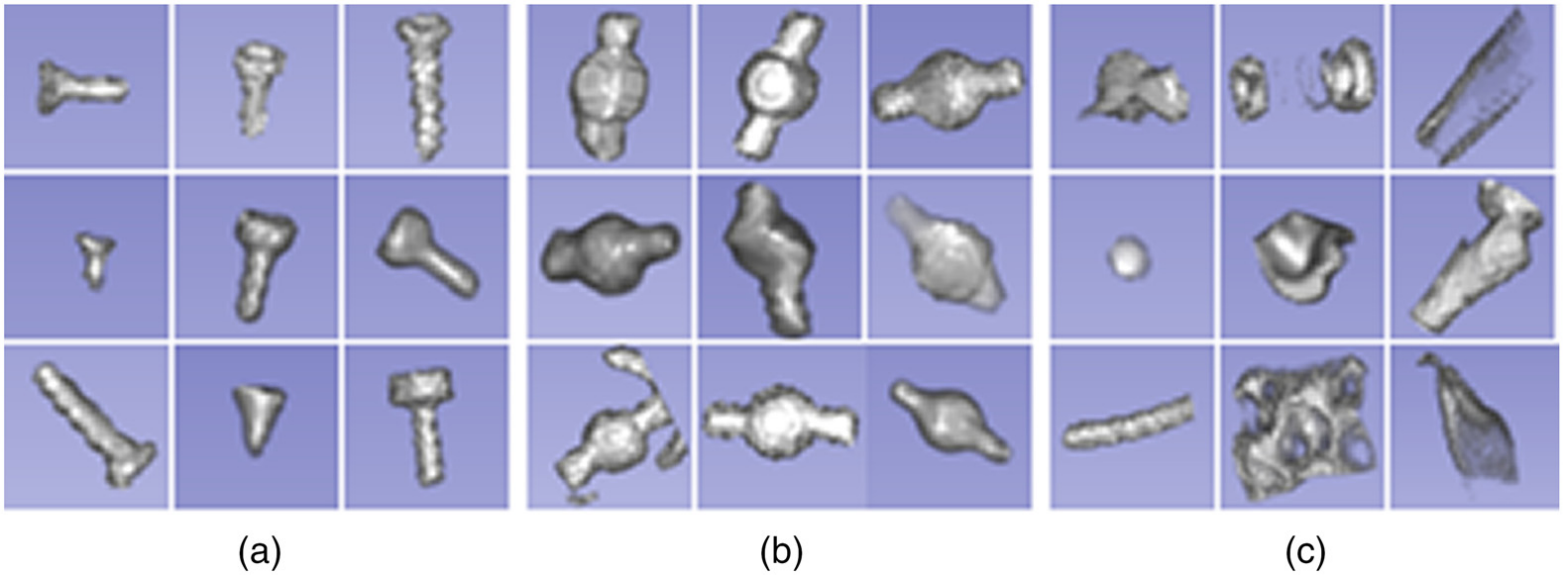
Example of automatically segmented samples in the test dataset. (a) Screws with different dimensions with the smallest and largest diameter 2 and 4 mm and length 3 and 8 mm. (b) Spherical fiducials with 4 mm in diameter and 8 mm in length. (c) Various background structures with material similar to fiducial markers.

The trained network is evaluated on unseen data containing 241 screws, 151 spherical fiducials, and 1550 background structures. In a similar manner as for the training, the test dataset was created from 43 CT images of 12 human anatomical specimen heads (64 screws, 24 spherical fiducials), nine porcine heads (43 spherical fiducials), 10 abdominal phantoms^40^ (60 spherical fiducials), and 12 skull phantoms (177 screws, 24 spherical fiducials). The images were acquired from at least two different scanners over a period of the last eleven years. The slice thickness varied from 0.4 mm up to 1 mm. To compensate for the impact of the fiducial material, the CTs were selected to have objects composed from copper, steel, and titanium (e.g., wires and holders). Segmented samples are shown in Fig. 11.

#### 3.1.2 Open-set evaluation

**Fig. 12:**
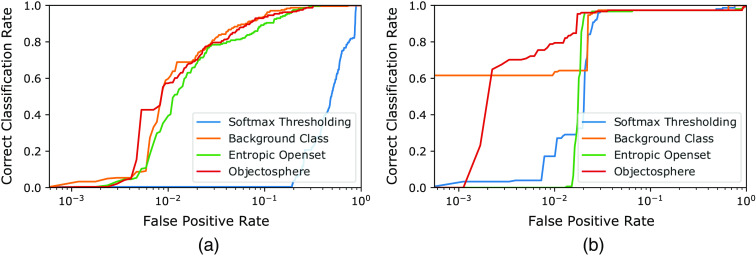
The OSCR curves with a logarithmic horizontal axis per a single multi-class classifier for (a) screw versus not-screw and (b) spherical fiducial versus not-spherical fiducial in the test dataset.

**Fig. 13:**
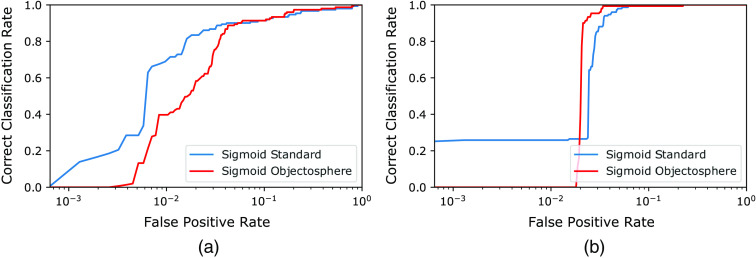
The OSCR curves with a logarithmic horizontal axis per a dedicated binary classifier for (a) screw and (b) spherical fiducial versus not-fiducial in the test dataset.

To select the best model in terms of open-set evaluation (separation of fiducial markers from other structures), we addressed the Open-Set Classification Rate (OSCR) metric proposed in Dhamija et al.^33^ This metric is suggested as more appropriate for open-set evaluation as its y axis is composed solely of known classes components, compared to, for example, precision-recall, which can be prone to data bias.^33^ The OSCR metric calculates, as a function of confidence thresholds, correct classification rate (CCR) and false positive rate (FPR). CCR is the fraction of known correctly recognized samples (true positives) and FPR is the fraction of the unknown samples recognized as the known class (false positives). Here, we look for the classifiers that outperform with higher CCRs at lower FPRs. Figures 12 and 13 show the inferences resulted from the trained models while Tables 1 and 2 gives the top CCRs at the lowest FPRs. For the trained multi-class models, fiducial classes were evaluated separately: first, screws were considered as knowns, whereas non-screw (spherical fiducial and background) as unknowns; second, spherical fiducials were considered as knowns, whereas non-spherical fiducial (screws and background) as unknowns.

**Table 1:** Experimentally determined CCR at the lowest FPR expressed as percentage for each classifier validated on screw versus not-screw and spherical fiducials versus not-spherical fiducial in the test dataset.

Classifier	Screw		Spherical	
	CCR (%)	FPR (%)	CCR (%)	FPR
Softmax Thresholding	75.1	63.1	96.7	3.0
Background Class	97.1	11.5	97.4	2.9
Entropic Openset	90.0	9.9	96.7	2.5
Objectosphere	93.0	8.0	96.0	1.9

**Table 2:** Experimentally determined CCR at the lowest FPR expressed as percentage for each binary classifier validated on fiducial versus not-fiducial in the test dataset.

Fiducial	Classifier	CCR (%)	FPR (%)	Balanced accuracy (%)
Screw	Sigmoid Standard	95.9	8.7	93.6
Screw	Sigmoid Objectosphere	93.0	6.5	93.3
Spherical	Sigmoid Standard	98.7	5.4	96.7
Spherical	Sigmoid Objectosphere	99.3	3.4	98.0

#### 3.1.3 Multi-class evaluation

Widely used measures for evaluating classifiers are sensitivity, specificity, and accuracy. In our open-set evaluation, it can be noticed that CCR quantifies the sensitivity and FPR complements the specificity of the proposed system:

$$\text{Sensitivity}=\frac{TP}{TP+FN},$$ (3)

$$\text{Specificity}=\frac{TN}{TN+FP},$$ (4)

where TP, FN, TN, and FP indicate true positive, false negative, true negative, and false positive counts, respectively. The standard accuracy metric is omitted because is sensitive to highly imbalanced dataset. Instead, a balanced accuracy metric can be used to compensate for imbalance:

$$\text{Balanced accuracy}=\frac{1}{2}(\text{Sensitivity}+\text{Specificity}).$$ (5)

In one-vs-one case, this metric is obtained straightforwardly and shown directly in Table 2. However, in multi-class case, the open-set evaluation only considers the performance of individual fiducial classes. To assess the quality of overall classification, an average of these measures calculated for each class i∈N where N=3 is represented:

$${\text{Balanced accuracy}}_{mc}=\frac{1}{2N}\overset{N}{\underset{i=1}{\sum }}({\text{Sensitivity}}_{i}+{\text{Specificity}}_{i})$$ (6)

The results are reported in Table 3.

**Table 3:** Experimentally determined mean values of sensitivity, specificity, and balanced accuracy in terms of overall multi-class classification.

Classifier	Sensitivity (%)	Specificity (%)	Balanced accuracy (%)
Softmax Thresholding	66.5	72.9	69.8
Background Class	93.0	94.7	93.8
Entropic Openset	91.1	93.8	92.5
Objectosphere	92.8	95.1	93.9

### 3.2 Evaluation of the localization method

#### 3.2.1 Testing dataset

Acquired testing data in CONRAD^27^ had specified parameters with 360° of rotations with an angular increment of 1 deg, detector image size 800×800 with an isotropic pixel size of 0.3 mm. The electron beam was simulated as a monochromatic beam with noise with 120 keV and 100,000 photons, following a Poisson distribution. Physical densities for air were used as a background medium, titanium as marker material, bone for skull, and brain for tissue. Datasets contained 15 markers with distribution and orientation being randomly chosen. Samples from virtual CTs are shown in Fig. 10. The projections were running on a Windows machine, CPU Intel Core i7-7700K 4.2 GHz, RAM 16 GB and reconstructed using GPU NVIDIA GeForce GTX 1050 (8 GB GPU RAM). Duration per scan projection was in the range 2 to 4 h while scan reconstruction was faster (∼15 to 30 min).

#### 3.2.2 FLE evaluation

The synthetic datasets were segmented and markers classified using the described methods. Following this, the mesh of the segmented marker was constructed and coregistered to the reference mesh using the aforementioned localization method (see Fig. 8). A rigid transformation applied on the defined fiducial point at the reference model was used to determine the fiducial point of the aligned marker in the image space. Since the image origin was moved to the center of the image, which corresponds to the phantom origin, the FLE was simply calculated as the Euclidean distance between the determined fiducial point in the image and the point in the virtual phantom for that marker.

The mean (±standard deviation) FLE results for 25 data sets with different markers and voxel size combinations are shown in Tables 4 and 5, with and without Laplacian smoothing prior to localization. Specific fiducials are encoded as F1 (screw $2\times 3\;{\mathrm{mm}}^{2}$); F2 (screw $3\times 3.75\;{\mathrm{mm}}^{2}$); F3 (screw $3\times 4.5\;{\mathrm{mm}}^{2}$); F4 (spherical marker $4\times 8\;{\mathrm{mm}}^{2}$); and F5 (spherical marker $3\times 6\;{\mathrm{mm}}^{2}$). Mean FLEs ranges from 14 to 177  μm, with spherical markers performing better.

**Table 4:** Experimentally determined FLE values in virtual CBCT images, with marker smoothing.

Voxel size (mm)	F1 (μm)	F2 (μm)	F3 (μm)	F4 (μm)	F5 (μm)
0.3×0.3×0.3	58(14)	83(36)	62(19)	16(7)	18(6)
0.3×0.3×0.6	79(29)	90(40)	64(26)	25(10)	26(12)
0.5×0.5×0.5	118(31)	72(27)	61(16)	41(22)	49(22)
0.5×0.5×0.6	120(44)	96(46)	84(30)	38(21)	48(25)
0.5×0.5×0.8	177(97)	120(53)	119(42)	49(25)	46(19)

**Table 5:** Experimentally determined FLE values in virtual CBCT images, without marker smoothing.

Voxel size (mm)	F1 (μm)	F2 (μm)	F3 (μm)	F4 (μm)	F5 (μm)
0.3×0.3×0.3	62(17)	92(31)	74(30)	15(6)	14(6)
0.3×0.3×0.6	77(32)	97(39)	71(33)	22(7)	33(26)
0.5×0.5×0.5	125(43)	96(26)	81(20)	30(18)	42(28)
0.5×0.5×0.6	115(42)	108(45)	100(42)	30(11)	43(26)
0.5×0.5×0.8	155(74)	122(54)	122(48)	41(16)	48(28)

**Fig. 14:**
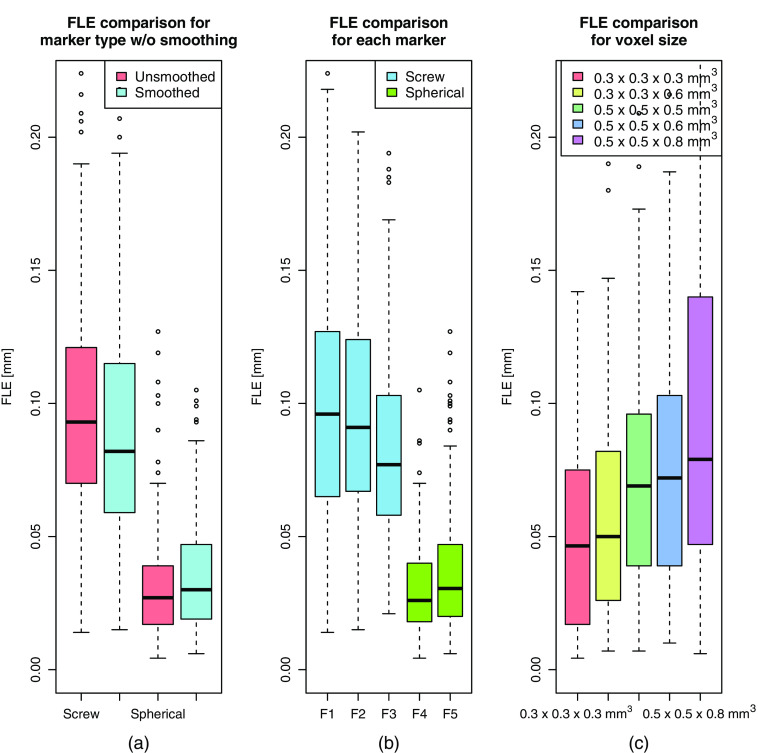
Boxplots of achieved FLEs in millimeters compared (a) for screw and spherical markers with and without (w/o) Laplacian smoothing; (b) for each marker independently in the datasets; and (c) for the recorded voxel sizes in the datasets.

Several Wilcoxon Signed Rank tests (two-sided, p-value<0.05) were used to determine for significant differences in FLEs. This is a non-parametric test as the FLEs were found to be not normally distributed (boxplot distributions in Fig. 14 and Shapiro-Wilk test, p-value<0.05). First, an overall evaluation is compared for screw and spherical marker FLEs in Table 4 against FLEs in Table 5. It was found that the screw median FLE with smoothing is significantly different compared to without smoothing. However, the absolute median difference is very small, 11  μm. Second, FLEs of each marker were compared against FLEs of the other markers in Table 4. No statistically significant difference was found between spherical markers F4 and F5. On the other hand, it was found between spherical markers and screws F1, F2, and F3. Also, FLEs of F3 screw were statistically significant to FLEs of F1 and F2 screws. Finally, FLEs for screw and spherical fiducials were compared for voxel sizes in Table 4. For screws, a trend toward significance was found for each voxel size combination except between $0.5\times 0.5\times 0.5\;{\mathrm{mm}}^{3}$ and $0.5\times 0.5\times 0.6\;{\mathrm{mm}}^{3}$. In contrast, for spherical fiducials, significance was found only for FLEs in $0.3\times 0.3\times 0.3\;{\mathrm{mm}}^{3}$ and $0.3\times 0.3\times 0.6\;{\mathrm{mm}}^{3}$ against others. These combinations are visualized with boxplots in Fig. 14.

## 4 Discussion

An algorithm for analysis of medical imaging data as presented in this work suffers from inherent limitations such as finite voxel size, acquisition artifacts, noise, background, and a selection of marker volume and shape. Therefore, it needs to be robust enough and carefully tested against these parameters. Among them, this research studied how finite voxel size, marker volume, and shape affect the FLE localization accuracy in particular. The results provide helpful insight into selecting these parameters for optimal performance. The FLE evaluation was performed with the proposed digital experiment that exploits CONRAD^27^-software framework to acquire realistic CBCT scans from virtual phantoms. Though it takes effort and time to construct virtual phantoms and generate virtual CTs, we conclude that it is straightforward and demands fewer physical resources.

As reported in the literature for physical phantoms,^20^^,^^41^ the lowest FLEs were obtained for datasets with smaller voxel size. The best achieved FLE mean and its standard deviation for a screw and spherical marker are 58(14)  μm and 14(6)  μm, respectively. Interestingly, the determined FLEs in images are better than previously achieved using physically acquired datasets.^25^^,^^26^ For instance, in similar marker dimensions and voxel sizes, Gerber et al.^25^ reports mean (±standard deviation) FLE 153 (61)  μm for screws, whereas Kobler et al.^26^ reports lowest FLE ∼40  μm for spherical fiducials. Possible explanations are due to eliminating contributing errors from physical scans and ground-truth measurements. Although it was not directly measured, we speculate that improved voxel-to-mesh generation^38^ could contribute to lower FLEs as well. It can also be noticed that the screw FLE is slightly lower when Laplacian smoothing^39^ is applied on the screw mesh prior to localization.

Spherical markers were superior to screws for both detection and localization assessments. It appears that the particular shape and larger size of the first compared to the second contributes to this difference. Further, significantly different FLEs were only found in smaller voxel sizes for spherical markers, whereas for screws in almost all used voxel sizes. This is an important finding as the voxel size is a clinical parameter that is directly related with the radiation dose delivered to the patient. Depending on the clinical question being asked, spherical markers demonstrate a lower trade-off between accuracy and radiation dose.

The OSCR metric,^33^ on the other hand, evaluated detection rates, with best CCR (at lowest FPR) achieved for 241 screw and 151 spherical fiducials 95.9% (8.7%) and 99.3% (3.4%) in binary classifiers and 93.0% (8.0%) and 96.0% (1.9%) in multi-class classifiers. In the latter case, the detection rate would be higher if one phantom image was excluded, which had all four spherical fiducials incorrectly recognized as screws by all four classifiers. Our detection rate with spherical fiducials is consistent with previously reported markers attached to the patient’s head in CT scans: Wang et al.^9^ perfectly identified 24 markers with 0% FPR; Wang and Song^15^ 69 over 75 markers with 0% FPR; Fattori et al.^17^ 211 over 233 (90.1%) markers with 0% FPR; and Bao et al.^20^ all 144 markers without reference to the false positives. In contrast, our evaluation was determined using a larger dataset of background structures, with fiducial material taken into the composition. To our knowledge, there is no prior work with automatically detecting surgical screws that we can directly compare with. One study worth mentioning achieves a true positive rate of 98.1% and an FPR of 4% for automated detection of cannulated screws (309 screws in total) used for treating intra-articular calcaneal fractures.^42^^,^^43^ Although our results are lower, one can argue that the higher detection rate of cannulated screws in image could be partially guaranteed by their larger volume (especially contributed by their long length that can be up to several centimeters compared to their diameter that is between 2 and 6 mm^42^) in contrast to fiducial screws that are required to be just a few millimeters for minimal invasive skull base surgery.^1^^,^^2^

This work improves and extends a traditional segmentation approach proposed by Gu and Peters^11^ for titanium screws and spherical fiducial. Moreover, as aforementioned, fiducial classification was evaluated with dedicated binary and single multi-class classifiers. As emphasized in early studies,^31^^–^^33^ the most inconsistent results were achieved using softmax thresholding, which incorrectly classified most of the background as screws while outperforming for spherical fiducial. The objectosphere classifier is an exciting approach and shows the potential to outperform others. Nevertheless, several iterations may be required to tune the hyperparameters. Previously this approach was employed only in 2D multi-class softmax models.^33^^,^^34^^,^^44^ We also demonstrated that, for the same hyperparameters, training the binary classifier on additional objectosphere loss could lead to better separate the two classes and improve the sigmoid scores. Nonetheless, our result must be cautiously interpreted and verified on other datasets. For our laboratory purposes, multi-class classification is functional since both fiducial types are embedded in the same image.^45^^,^^46^ However, apart from this scenario and more importantly, a single fiducial type in the clinical setting is more commonly used per medical procedure. Therefore, it would make sense only to utilize a binary classifier, which outperforms demonstrated multi-class classifiers into the bargain.

Using CNNs enabled to model marker image representation three-dimensionally, hierarchically and on a higher feature level. It also standardizes the detection method, which in the future can be extended for other types of fiducial markers as well. The disadvantage is that they are challenging to train, require high computational resources, and large datasets. Though, once trained, the predictions are very fast. To avoid biased results, our deep network is tested on unseen data containing most of the available data. This is left to train the network on a small dataset (mainly constructed from phantoms), which is extensively enlarged with rigid transformations for data augmentation to improve generalization and avoid overfittings to any special pattern. We speculate that improving training datasets and reducing data augmentation can help learn better detailed features from segmented objects, which could lead to better detection accuracy.

Although the algorithm works well in our laboratory setting, one limitation of the proposed three-step approach is that the whole pipeline is extended and subjected to changes in context such as adaption of pre-processing steps for thresholds and noise reduction. Hence, an outlook for future upgrades is to expand the 3D CNN also for the task of marker segmentation.^47^ Another alternative to our classification approach is to use the R-CNNs for direct object detection.^48^^–^^50^ In addition, the proposed CNN architecture can be modified to directly approximate the location and orientation of the markers using additional numerical coordinate regression layers.^51^ This would allow a single step forward registration or at least would provide a good initial value, which could eliminate or reduce the computation time required currently by the ICP step.

## 5 Conclusions

In summary, the presented algorithm is fully automatizing detection and localization of titanium screw and spherical fiducials with high accuracy for different marker sizes and resolutions. Effectively this will lead to reduced resources and errors introduced by human interactions in high-accuracy frameless surgery. The presented synthetic experiment can simplify FLE estimation and might need fewer resources compared to physical acquisition.
